# RF Welding of Dielectric Lossless Foam Particles by the Application of a Dielectric Heatable Coating with High Recycling Potential

**DOI:** 10.3390/polym15193950

**Published:** 2023-09-29

**Authors:** Kevin Schneider, Tobias Kleffel, Dietmar Drummer

**Affiliations:** Institute of Polymer Technology, University of Erlangen-Nuremberg, Am Weichselgarten 10, 91058 Erlangen, Germany

**Keywords:** high-frequency welding, plastic foams, recyclability, water-soluble, coating, dielectric loss, plasticizer

## Abstract

Due to its chemical structure and the resulting dielectric properties, the processing of the commonly used particle foam material, expanded polypropylene (ePP), is limited. Processing within the radio-frequency welding process is therefore only possible with the use of processing aids. In this paper, a new approach for the use of a solid and dielectric heatable coating for the production of three-dimensional welded components out of ePP is presented. For this purpose, three different types of water-soluble polymer polyvinyl alcohol (PVA) were analyzed as potential coating materials. The thermal and dielectric properties of the coating were further adjusted by a modification with glycerol. The maximum amount of glycerol tested was 25% by volume. It influences both the temperature development in the radio-frequency (RF) welding process as well as the adhesive bond between the ePP foam particles. It is shown that the 120 °C approach in the RF welding process resulted in a cohesive bond between the coating layers. In this way, bonded plates can be produced. In mechanical tests with compression of 20%, the manufactured plates show sufficient load capacity. Furthermore, it can be shown that a separation of PVA and ePP by type, and thereby a separation of the foam particles, is possible with the use of hot water. This might open a new way for recycling of particle foams.

## 1. Introduction

Components made of foamed plastics combine a number of advantageous properties, such as low density in the range of 15–120 g/L [1], good sound insulation, as well as low thermal conductivity [2]. As a result, they are widely used in the field of packaging [3], cushioning [4] and insulating materials [5]. Foamed components are mainly produced by extrusion and by welding pre-expanded foam particles. While simple geometries such as sheets or strands are produced in extrusion, the geometric freedom is significantly higher when foamed particles are welded together. The advantage of welding pre-expanded particles over foam extrusion is a uniform cell structure within the entire component [6]. The current standard process for manufacturing three-dimensional foam components from foam particles is the steam chest molding (SCM) process. In this process, the welding of the individual foam particles is achieved by heating them with hot steam. A typical machine can use up to 13,200 L of water daily [7]. Due to the high heat capacity of water, the production of steam is inevitably associated with high amounts of energy. Usually, the steam generator and the processing machines are spatially separated, resulting in additional losses caused by the piping. Furthermore, a high amount of the converted heat is used to heat the metal mold [8], which needs to be cooled for the next processing cycle. Accordingly, only a small part of the energy expended is used for welding the foam component, which is why the efficiency of the process can be classified as low.

A previous study [9] investigated a novel approach for welding three-dimensional foam components through the radio-frequency (RF) welding process, which is usually used for the processing of thin films [10] and layered fabrics [11]. The heating of a particle foam made of expanded thermoplastic polyurethane (eTPU) and the used tools based on polyethylene terephthalate (PET) and polyoxymethylene (POM) were analyzed [9]. It could be shown that the used eTPU beads could be welded to three-dimensional plates by the RF process. With additional modifications to the tool’s thermal conductivity, the cycle time could be reduced by over 30%.

The decisive factor for the interaction and heating of plastics with the electromagnetic field is the polarity of the material used. Permittivity is a characteristic value that indicates how well the dipoles can align with the external electrical field. The force of the altering external electric field causes a repeatedly changing alignment of the dipoles present in the material. The resulting internal friction causes the material to heat up [12]. The proportion of converted electrical energy into thermal energy can be described by the dielectric loss factor. It is influenced by the chemical structure and the presence of dipoles in the polymer [13], the prevailing temperature, as well as the frequency of the applied field. It may also be influenced by newly introduced fillers [14]. However, if a plastic material does not possess any permanent dipoles, its interaction with an electric field is also limited. Consequently, it is not feasible to achieve sufficient heating for welding through dielectric heating. This behavior is also observed in widely used particle foam materials such as expanded polystyrene (ePS) and expanded polypropylene (ePP). The processing of particle foams based on these plastics using the RF-welding method is therefore not possible without processing aids. One possible approach is the utilization of water as a process aid. However, this is accompanied by additional challenges such as electrical insulation and the pressure-tight design of the tooling technology used. These problems could be circumvented with another previously proposed theoretical approach [15]. The non-heatable foam particles could be modified with a coating with a high dielectric loss factor to enable a local heating on the particle surface. Due to its dielectric loss factor of up to 250 mU, polyvinyl alcohol (PVA) should be an appropriate coating material, which can also be optimized by the use of plasticizers such as glycerol. However, it is not known how such core-shell particles work in practice and which component properties can be achieved. Thus, this paper is focused on a more detailed investigation of the dielectric properties of the used coating material, the corresponding heat generation in the RF welding process, and the mechanical properties of the generated plate samples.

## 2. Materials and Methods

### 2.1. Materials

Due to their frequent use in the field of packaging or insulation and, at the same time, low material costs, spherical-shaped foam particles based on expanded polypropylene (ePP) with the type code Neopolen P 9255 (BASF SE, Ludwigshafen, Germany) were used as base material. According to the data sheet, the particle size averages between 1.5 and 4 mm in diameter. The bulk density is given at 50–60 g/L, while the mean particle weight ranges between 1.0 and 1.4 g [16]. As the coating material, the water-soluble polymer polyvinyl alcohol (PVA) was selected. This polymer is manufactured by the saponification of polyvinyl acetate. Resulting in the functionalization of every second carbon atom of the polymer main chain by either an alcohol or an acetate side-group, resulting in comparable high permittivity and dielectric loss factor and thus good dielectric heating. Due to the combination of possible re-drying as a coating on the surface of the foam particles and, at the same time, good dielectric heatability, PVA is assumed to be a suitable coating material for the intended application. Since the degree of saponification and, with it, the number of OH- side groups influence both the dielectric and thermal properties of the polymer, three different types (Poval 2-98, Poval 3-85, and Poval 5-74) from Kuraray Europe GmbH (Frankfurt am Main, Germany) were investigated. The second number of the type name indicates the number of OH- side groups in percentage. Hence, 2-98 has the highest amount, while Poval 5-74 has the fewest. All three PVAs were provided in the form of granules. The material properties of PVA can further be modified by the use of different plasticizers. Within the scope of this work, glycerol type Glycerin 1000 from Merck KGaA (Darmstadt, Germany) was used. Due to its chemical structure with multiple OH- groups, glycerol influences both the dielectric and thermal properties of the coating.

### 2.2. Material Modification and Sample Manufacturing for Material Characterization

#### 2.2.1. Aqueous PVA Solutions

An aqueous solution was used as the basis for the coating process but also for dielectric and thermal characterization. This solution contains either PVA or PVA–glycerol mixtures. All produced solutions had a proportion of dissolved substances of 10 vol-% in total. The influence of the modification with glycerol was investigated only for Poval 3-85 since it is representative of the other types as well. The amount of glycerol was varied between 0 and 2.5% by volume based on the total mixture. This results in PVA/glycerol formulations from 100/0 up to 75/25 without water. The compositions of the liquid solutions and the resulting PVA/glycerol formulations are given in Table 1.

For the production of an aqueous solution, the required amounts of PVA and water were given into a beaker as a first step. The mixture was heated to 80 °C and stirred continuously for 2 h until all PVA particles were dissolved. After the mixture cooled down to room temperature, the evaporated amount of water was refilled, and the amount of glycerol, if needed, was added to the solution. Afterwards, the solution was stirred for an additional 5 min to achieve a homogeneous mixture. 

#### 2.2.2. Production of PVA-Glycerol Granule

For the characterization of the dielectric and thermal properties, a solid material is required. For this purpose, the aqueous solutions of the different PVA/glycerol formulations were poured into silicon molds. Afterwards, the water was evaporated at 90 °C for at least 48 h in a convection oven type Heratherm OMH750 from Thermo Electron LED GmbH (Langenselbold, Germany). After the drying process was finished, the samples were demolded and shredded into free-flowing granules. 

#### 2.2.3. Production of Sample Plates in Injection Molding

For the dielectric characterization of all used materials (ePP, modified, and unmodified PVA), solid plates with plane-parallel surfaces are required. Therefore, 50 × 50 × 2 mm^3^ plates were produced by injection molding on an ERGOtech 25-80 from Sumitomo (SHI) Demag Plastics Machinery GmbH (Schwaig, Germany). Since PVA is hydrophilic, the granules of the unmodified materials as well as the PVA–glycerol combinations were dried before processing for 8 h at 90 °C. The main process parameters are shown in Table 2. The parameters were kept constant for all PVA-based materials.

#### 2.2.4. Particle Coating

The coating of the ePP foam particles with the aqueous PVA solutions was performed in a self-developed small-scale drum coating machine. The drum had a length of 300 mm and a diameter of 270 mm. A grid was used as the surface of the drum. The grid is characterized by square holes with a size of 1 × 1 mm^2^, which are separated by 1 mm-thick bars. The rotation speed of the drum was set to 40 RPM. Within one coating operation, 110 g of ePP particles were processed. The PVA (-glycerol) solution was applied in multiple cycles to the ePP particles by a two-substance nozzle. For each cycle, the volume of applied liquid was set to 10 mL. Due to a constant air stream through the drum, the water in the solution vaporized within 300 s. Then the next cycle started. In this way, a coating of back-dried PVA could be created on the particle surface. To generate a sufficient PVA-layer thickness, the coating process was repeated eight times in total. 

### 2.3. Dielectric Properties

The dielectric material permittivity and dielectric loss factor are decisive for the dielectric heating of a material inside the RF-heating process. Therefore, the permittivity and dielectric loss factor of the injection-molded samples in a dry state were measured with a Keysight E4990A Impedance Analyzer from Keysight Technologies, Inc. (Santa Rosa, CA, USA) in combination with a Keysight 16451B Dielectric Test Fixture. Both the impedance analyzer and test fixture are specified for frequencies up to 30 MHz. In accordance with DIN-EN 62631-2-1 [17], the dielectric measurements were performed at a frequency of 27.12 MHz. In the following, the permittivity will be given without units, while the loss factor will be specified by milliunits (mU). Since the dielectric material properties are temperature dependent, the measurements were also carried out at elevated temperatures. To do so, the test fixture and the samples were placed inside a convection oven of the type Heratherm OMH 100 from Thermo Fisher Scientific Inc. (Waltham, MA, USA). The investigated temperature range of 20 to 100 °C was divided into 10 °C steps. To avoid damage to the test fixture caused by high temperatures, the maximum temperature was set to 100 °C.

### 2.4. Differential Scanning Calorimetry (DSC)

For the investigation of the melting behavior of the used materials, which is important for the joining of the particles, differential scanning calorimetry (DSC) measurements were performed using a Q2000 from TA-Instruments Inc. (New Castle, DE, USA). For all materials, the heating and cooling rates were kept constant at 10 K/min. All tests were started at a temperature of 20 °C. The maximum temperature was set to 250 °C. Exemplary for the influence of glycerol on the thermal behavior of PVA, all five modified materials based on Poval 3-85 were measured as well. In order to show a material behavior as similar as possible to the processing in the RF machine, the first melting cycle was analyzed.

### 2.5. RF-Welding

**Figure 1:**
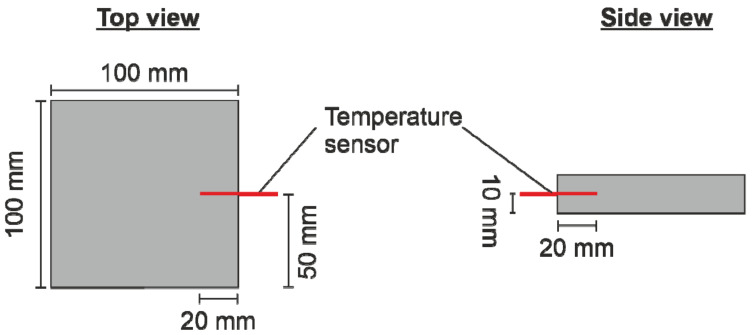
Position of the temperature sensor inside the plate geometry.

By heating and welding the coated ePP particles inside an altering electric field, the concept of processability due to surface modification should be verified. The welding trials were performed on an RF test plant based on a WAVE FOAMER machine from Kurtz Holding GmbH & Co. Beteiligungs KG (Kreutzwertheim-Weibelbach, Germany). The altering electric field used had a frequency of 27.12 MHz. The used mold was made out of polytetrafluoroethylene (PTFE), which shows no significant intrinsic heating inside the electric field. The cavity inside the mold was 100 × 100 × 20 mm^3^ in size. The electrode distance was 47 mm. The temperatures inside the foam sample were monitored with the fiber-optical temperature measurement system FOTEMP MK 19″ from Weidmann Technologies Deutschland GmbH (Dresden, Germany). Since the highest heating rates will be achieved in the center of the sample, the tip of the sensor was placed according to Figure 1 at the half sample height of 10 mm. All materials were heated with a voltage of 6 kV. The abortion criterion for the welding process was either a sample core temperature of 120 °C to avoid a complete melting of the sample or a maximum heating time of 200 s. The amount of particles filled into the mold was kept constant at 16 g, resulting in a sample density of 80 g/l.

### 2.6. Optical Analysis

To investigate the way in which the foam particles are bonded together, the produced plates were manually broken by bending. Afterwards, the fracture surface was observed by a stereo-microscope type Zeiss Axio Zoom.V16 from Carl Zeiss AG (Oberkochen, Germany). The final images are composed of multiple pictures with a height variation of 13 µm. Additionally, the fractured surface was investigated with a scanning electron microscope (SEM) Gemini Ultra Plus from Carl Zeiss AG (Oberkochen, Germany). By using energy-dispersive X-ray spectroscopy (EDX), a differentiation of the particle surface and an applied coating could be made. Microscopic images of polished cross-sections could not be prepared because the coating would have dissolved during water-cooled grinding. Due to the high flexibility of the foam particles, it was not possible to take thin sections from the samples.

### 2.7. Compression Test

The mechanical compressibility of selected plates with optically stable connections between individual particles was tested in accordance with DIN EN ISO 3386-1 [18]. Thereby, a circular stamp with a diameter of 60 mm was pressed into the samples. The initial height of the samples was determined by the punch spacing, at which an initial force of 20 N was measured. Prior to the actual measurement, the samples were compressed three times by 20% of their initial height. Afterwards, the new sample height was measured again according to the required force of 20 N. The final compression by 20% related to the initial sample height was recorded and evaluated. 

### 2.8. Recycling Test

In order to investigate the separation of the welded joint based on the PVA coating, recycling tests were carried out with a welded plate. First, the weight of the plate was determined. Then, 400 mL of water at 80 °C was placed in a beaker, and the plate was added. The solution was continuously stirred at 400 rpm for 60 min, and the temperature was kept constant at 80 °C using a hot plate. To ensure complete wetting of the plate, it was pressed under water with the aid of a grid. Finally, the water was poured off, and the remaining sample was dried and measured gravimetrically again. In addition, the degree of particle separation was determined.

## 3. Results

### 3.1. Dielectric Properties

**Figure 2:**
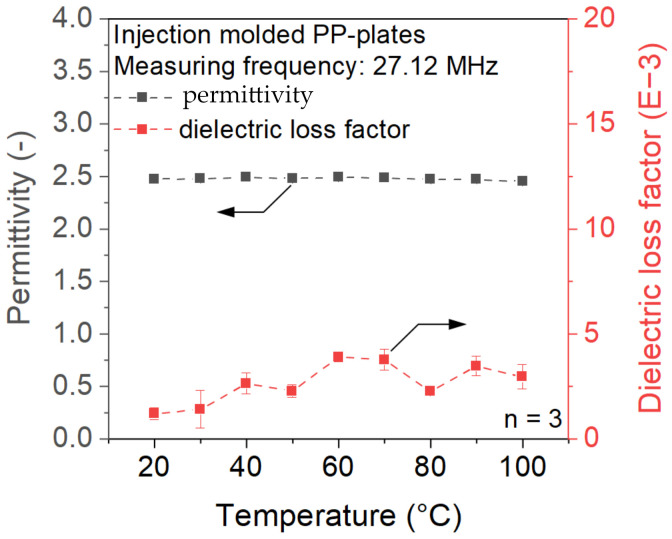
Dielectric properties of PP at a measurement frequency of 27.12 MHz.

In the first step, the temperature-dependent dielectric properties of PP and PVA-based materials were investigated. As displayed in Figure 2, the permittivity and dielectric loss factor of PP show no significant temperature dependence. The material permittivity remains in the range of 2.4 to 2.5, while the dielectric loss factor constantly stays below 5 mU. According to Abele [19], a dielectric loss factor of at least 10 mU is required to achieve sufficient heating inside the electrical RF field. Due to the encapsulated air inside the beads, the ePP particles will show even lower dielectric values compared to Figure 2. It can therefore be assumed that heating ePP within the electric field will not result in sufficient temperature development to achieve a welded joint.

**Figure 3:**
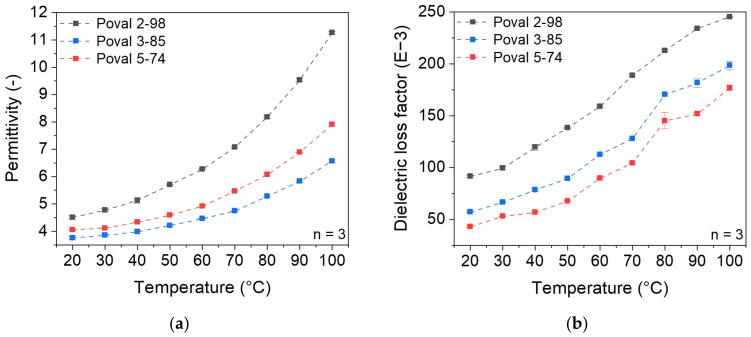
Temperature-dependent dielectric properties of PVA materials with varying degrees of saponification at a testing frequency of 27.12 MHz; (**a**) permittivity; (**b**) dielectric loss factor.

In contrast to nonpolar PP, the investigated coating materials based on different types of PVA show higher dielectric properties. Additionally, a strong temperature dependency of both permittivity and dielectric loss factor is detectable, as displayed in Figure 3. Both properties increase with rising temperatures. An exponential increase can be observed in the permittivity, while the increase in the dielectric loss factor is approximately linear. 

For the unmodified PVA materials, the permittivity and dielectric loss factor values show significant differences depending on the PVA type. Poval 2-98 with the highest percentage of OH- side groups, which describes the saponification degree, shows the highest values of permittivity and dielectric loss factor, while the least saponified PVA (Poval 5-74) has the lowest values. Poval 3-85 with an intermediate degree of OH- side groups ranges in between. At room temperature, permittivity values of 3.75 (Poval 5-74), 4.05 (Poval 3-85), and 4.51 (Poval 2-98) were measured. Due to the described exponential increase in permittivity, the difference in the measured values at 100 °C is clearly higher. So Poval 2-98 shows a maximum permittivity of 11.25, while Poval 5-74 only reaches 6.57. Regarding the dielectric loss factor, the curve slope of all three PVA materials is comparable. The degree of saponification only influences the vertical offset. At room temperature, the value rises with increasing amounts of OH- side groups. For Poval 5-74, a dielectric loss factor of 43.12 mU is measured. Poval 3-85 reaches a loss factor of 57.10 mU, and Poval 2-98 91.50 mU. The highest overall loss factor was 245.33 mU for Poval 2-98 at 100 °C.

The temperature dependency of both permittivity and dielectric loss factor can be explained due to the higher mobility at the molecular level. With increasing temperatures, the dipoles of the polymeric chain can align more easily with the external electrical field. At the same time, the number of dipoles is related to the PVA’s degree of saponification.

**Figure 4:**
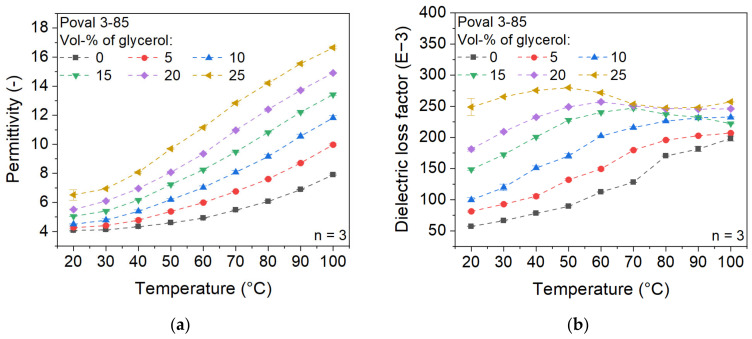
Influence of glycerol on the dielectric properties of Poval 3-85; (**a**) permittivity; (**b**) dielectric loss factor.

The influence of the added plasticizer on the dielectric properties of Poval 3-85 is displayed in Figure 4. In relation to the permittivity, the glycerol used causes an increase in the value, which manifests as a vertical shift to higher values at the same temperature. While unmodified Poval 3-85 has a permittivity of 4.05 at 20 °C, the value is raised to 6.51 for Poval 3-85 with 25 vol-% glycerol. When the measuring temperature is increased, the unmodified PVA material shows an exponential increase in permittivity. The rising proportion of glycerol causes a constant change to become a more linear increase. In addition, glycerol causes a slight increase in the slope, resulting in a higher deviation of the various materials at 100 °C compared to 20 °C. At 100 °C, the permittivity ranges from 7.90 to 16.65 due to the influence of the amount of glycerol used.

Regarding the dielectric loss factor, the curves of the varying glycerol contents are different. For the unmodified PVA sample, the values increase with rising temperature within the investigated temperature range. With increasing glycerol content, the maximum increases slightly. At the same time, it shifts to lower temperatures. In addition, after reaching the maximum value, a decrease to an approximately constant value can be observed at high glycerol contents. However, the dielectric loss factor of the glycerol-modified PVA is always higher, independent of the glycerol content and the temperature. 

The material behavior described can be explained by the plasticizing effect of glycerol on PVA. As the proportion of plasticizer increases, the mobility of the molecules also increases, which is reflected in enhanced dielectric properties. The described effects of glycerol are given for all three investigated PVA types. Therefore, a wide range of possible dielectric properties can be achieved by the combination of PVA and glycerol. 

### 3.2. DSC

**Figure 5:**
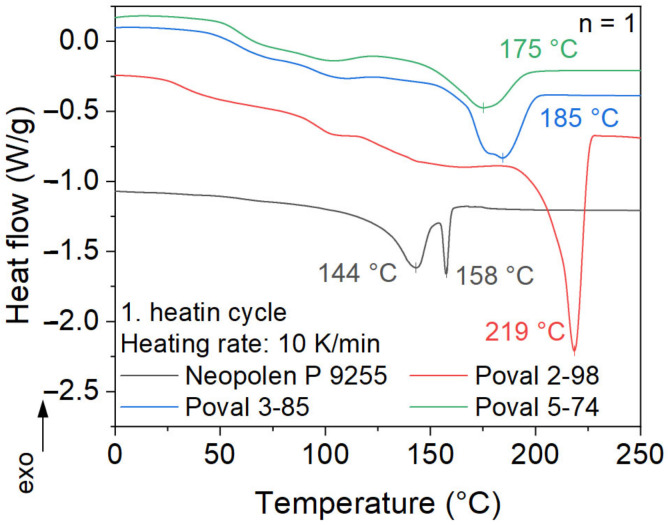
DSC analysis of first melting.

The melting behavior of ePP and all unmodified PVA materials is shown in Figure 5. For the different PVA materials, a clear correlation between peak melting temperature and the degree of saponification is visible. Poval 5-74 shows the lowest melting peak temperature of 175 °C. With an increasing amount of OH- side groups, the melting temperature shifts to higher temperatures. This results in a variation of 42 °C between Poval 5-74 and Poval 2-98. The increased amount of OH- side groups in Poval 2-98 results in a much sharper and lower melting peak compared to the other PVAs. In addition, the melting enthalpy increases with a rising degree of saponification. This results in a higher required amount of total energy to melt Poval 2-98 compared to the other PVAs tested. 

For the analyzed ePP, a double melting peak is measured. This behavior is typical for autoclave-foamed PP particles [1]. The two melting peak temperatures are 143 °C and 158 °C. For the used heating rate of 10 K/min, the first peak shows a broad melting temperature range. Its onset temperature is around 120 °C. Additionally, higher heating rates may result in a slight shift of the first peak to even higher temperatures. Both peaks are related to crystalline phases in α modification with various degrees of perfection [1]. In the commonly used manufacturing process of steam chest molding, heating to a temperature in between the two peaks is targeted [20]. 

**Figure 6:**
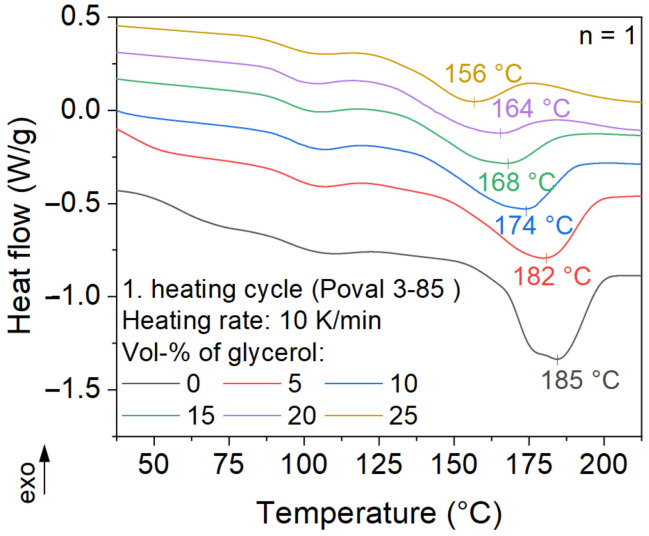
Influence of plasticizer on the melting behavior of Poval 3-85.

The modification of PVA with glycerol results in a shift of the melting peak to lower temperatures, as displayed in Figure 6. While the unmodified Poval 3-85 has a melting peak at 185 °C, the peak temperature is lowered by nearly 30 °C to 156 °C by a glycerol content of 25 vol-%. At the same time, the melting enthalpy decreases with increasing glycerol content. On the one hand, this is related to the substitution of PVA by the plasticizer, resulting in an overall lower amount of crystallized material. On the other hand, a reduction in the intermolecular interaction of the PVA chains may be assumed. Thus, the modification of PVA with glycerol results in a lower total temperature and, at the same time, a reduced amount of energy consumed to transfer the material from a crystalline to a molten state. Both changes in melting behavior are advantageous regarding reduced energy consumption. This results in shorter possible cycle times and lower energy consumption. 

### 3.3. Heating Trials

**Figure 7:**
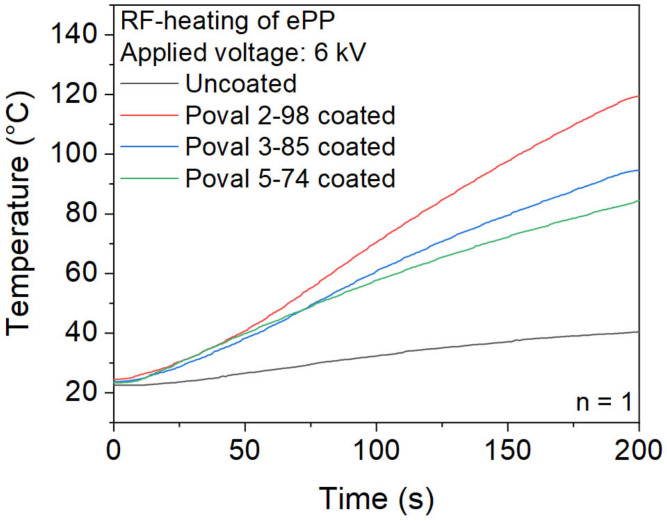
Heating behavior of uncoated and coated ePP particles with unmodified PVA.

First, a comparison of uncoated ePP particles and glycerol-free PVA coatings is displayed in Figure 7. It becomes clear that heating uncoated ePP foam particles for a maximum heating time of 200 s at a voltage of 6 kV results in a negligible temperature rise. The reached temperature of 40.4 °C is far from the required temperatures to achieve a partial melting of the ePP particles required for the welding. The reduced heating behavior correlates with the measured dielectric loss factor of the ePP base material.

In contrast, the ePP particles with a plasticizer-free PVA coating show significantly higher heating rates. However, there is a clear difference between the used PVA types. Since the parameters of the coating process are kept constant for all materials, the heating rates can also be correlated to the dielectric loss factors. Therefore, the coating based on Poval 2-98 with the highest dielectric loss factor results in the highest heating rate. The abort criterion of 120 °C was reached after a heating time of 200 s. Both the Poval 3-85 and Poval 5-74 coatings did not reach the 120 °C abortion criterion during the heating time of 200 s. However, the maximum temperatures reached differ. After 200 s of heating, a maximum temperature of 95 °C was measured for the Poval 3-85-coated foam particles. At the same time, the Poval 5-74-coated material was heated to a maximum temperature of 84 °C.

**Figure 8:**
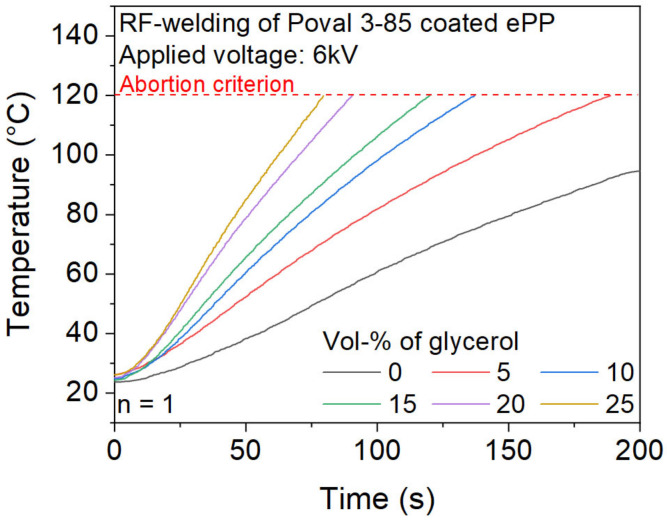
Influence of glycerol content of the Poval 3-85 coating on the heating behavior during RF-heating.

The increased values of the dielectric loss factor of Poval 3-85 due to the modification with glycerol are also reflected in the results of the temperature measurement inside the foam sample during the RF heating process, as displayed in Figure 8. The modification with glycerol results in a significant increase in the heating rate due to an increased dielectric loss factor. The effect is consistent for the investigated glycerol content from 0 to 25 vol-%. While unmodified Poval 3 85 only achieves 95 °C within 200 s of heating, the addition of 5 vol-% of glycerol leads to a noticeable higher heating rate. This results in a heating time of 189 s until the upper temperature criterion of 120 °C is reached. With further increases in the glycerol content, the time to reach the 120 °C limit continuously decreases. For Poval 3-85 with 25 vol-% glycerol, heating to 120 °C is finished within 80 s. 

The heating trials presented in Figure 7 and Figure 8 resulted in form-stable plates. Although the temperatures reached are too low to ensure sufficient melting of ePP or the coating material according to the previously performed DSC. A possible explanation could be locally higher temperatures at the contact areas of individual particles. The difference in measured and appearing temperatures might be caused by the comparably high mass of the temperature sensor in combination with the low thermal mass of the heatable coating material. Since the used mold is not tempered and has a comparable high mass, its temperature remains at values between 30 and 45 °C during the welding process. Due to the conduction of heat from the foam to the mold, the plate stays rather cold on the surface. This causes a bumpy appearance on the surface.

### 3.4. Optical Analysis of Particle Coherence

**Figure 9:**
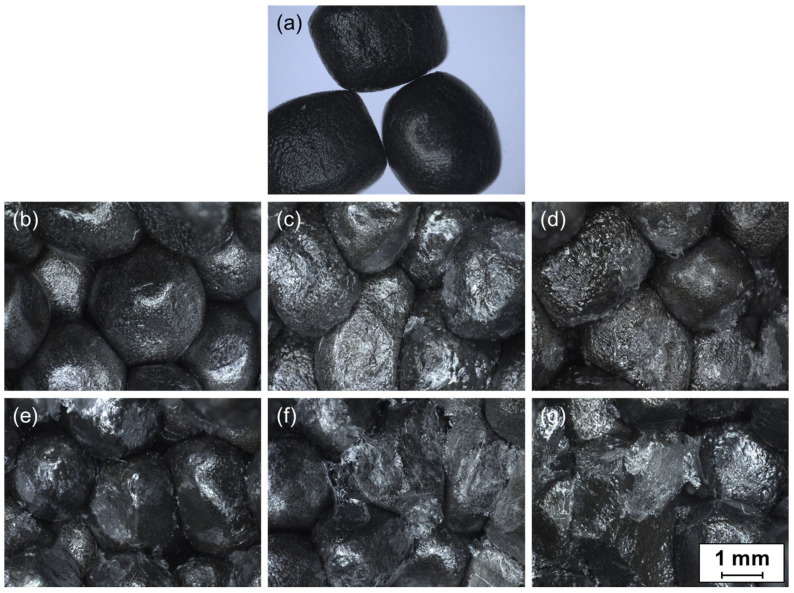
Stereomicroscopy images of reference ePP particles (**a**) and fracture surfaces of ePP-coated plates; (**b**) Poval 2-98—0% glycerol; (**c**) Poval 3-85—0% glycerol; (**d**) Poval 5-74—0% glycerol; (**e**) Poval 3-85—5% glycerol; (**f**) Poval 3-85—15% glycerol; (**g**) Poval 3-85—25% glycerol.

The microscopic images of unmodified ePP particles as well as of fracture surfaces from the inside of the welded plates for the three unmodified PVA and three Poval 3-85 modified coatings are shown in Figure 9. In comparison to the unmodified particles, a shiny white finish is visible for all coated samples. The representative stereomicroscope images show that, independent of the coating material, the achieved temperatures were too low to melt the ePP material noticeably. The particles inside the sample appear as individual volumes separated from each other. No welding of the ePP foam particles was observed in any of the samples tested. The particles show a deformed appearance compared to their original spherical shape due to the compression force during the heating process. 

In comparative observation of the unmodified PVA materials, hardly any additional material is visible on the particle surfaces for the Poval 2-98-coated foam beads (Figure 9b). Only in a few spots, a white to transparent material can be identified. A connection between the particles is not visually recognizable. With a reduction in the PVA saponification level, the amount of transparent to whitish-colored material on the particle surface increases. For Poval 5-74 (Figure 9d), the largest amount of protruding coating material is visible. In some areas, the coating seems to connect the individual particles with each other. In addition, the remains of the torn-off coatings seem to be recognizable. This might be connected with the significantly lower melting temperature of the PVA type. 

The addition of glycerol to the PVA coating results in a higher amount of formed links between the foam particles, as shown in Figure 9c compared to Figure 9e–g for Poval 3-85. This could be due to the lower melting temperatures and the required amount of energy to melt the materials. At the same time, the increased heating ramps might lead to higher local temperatures at the contact area of individual particles.

**Figure 10:**
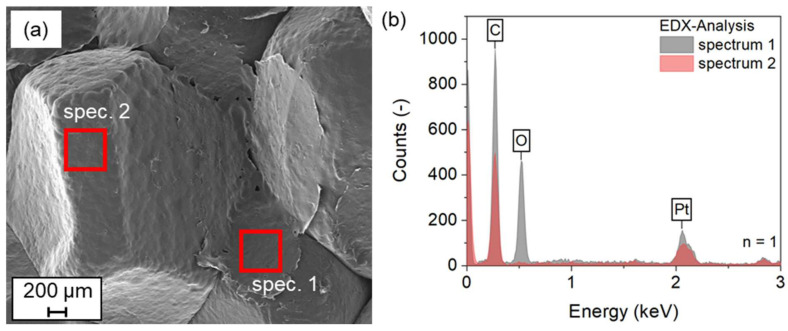
SEM+EDX investigation of fracture surfaces of ePP with Poval 3-85 + 20 vol-% glycerol coating; (**a**) SEM image with marked areas of the EDX investigation; (**b**) EDX spectra measured.

As shown in Figure 10a, the SEM image allows a detailed optical analysis of the fracture surface. It can be seen that in some areas there is a continuous layer between the particles. However, this is not the case for all areas. Rather, it can be assumed that the coating is made of a particle that has been removed from the surface. At the same time, this means that a cohesive bond has formed between the coatings of the particles in contact. The formation of a network of coatings is the result. The fracture of the specimen, on the other hand, occurs along the interface between particles and coatings. This assumption can also be confirmed by EDX analysis. Oxygen can be detected in spectrum 1, which corresponds to the coating left behind by the torn-off particle. Spectrum 2, on the other hand, does not show any oxygen. Here, the surface of the ePP particle can be assumed. Since the temperatures required to melt the PVA material are above the measured 120 °C, the hypothesis of the occurrence of higher local temperatures at the contact area between the particles is supported. The higher temperatures could be achieved by the higher local amount of PVA material, with its high dielectric loss factor.

### 3.5. Compression Behavior

**Figure 11:**
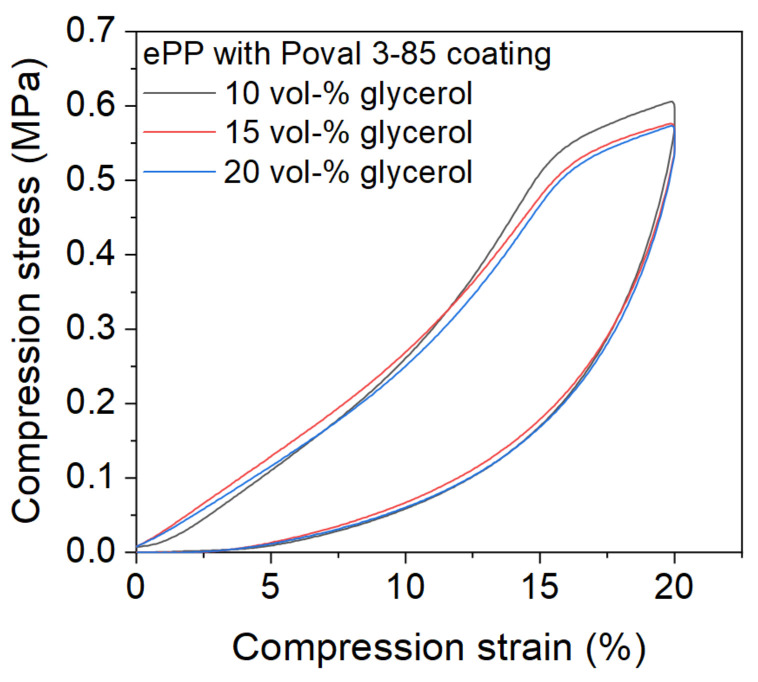
Mechanical properties of RF-welded-coated ePP samples with a thickness of 20 mm in compression.

Based on the optically stable connections between the individual particles, the scope of the investigation was narrowed down to Poval 3-85 in combination with 10, 15, and 20 vol-% glycerol. Figure 11 shows the compression behavior for the compression of up to 20% of the initial sample height after three initial compression cycles were preceded. Although the connection between the foam particles is based only on the PVA coating material, none of the samples were damaged due to the mechanical load. The curves initially show an almost linear increase in compressive stress. At 15% compression strain, a reduction in the slope can be observed. This behavior is due to the three preceding load cycles, which already slightly pre-compress the material. Subsequently, the value continues to increase with a reduced slope. The maximum value is reached at a compression strain of 20% for all three materials. All tested coating combinations resulted in comparable load behavior. In addition, the maximum compression stresses were similar. 

Based on the results presented in this paper, it can be shown that the investigated approach of particle connection ensures sufficient mechanical stability in compression. In future investigations, a comparison to steam chest-molded samples is planned. Further investigations regarding tensile strength and rebound behavior are considered.

### 3.6. Recyclability of Welded Samples

The recycling tests of all Poval 3-85-based coatings show promising results, which are presented in the following. Independent of the amount of glycerol in the PVA coating, the plates were completely separated into individual ePP beads within the given time of 60 min. After the loose ePP beads were dried, the mass of the particles was lowered by 6.4% ± 0.3% compared to the mass of the plate. The reduced mass can be attributed to the dissolution of the PVA coating. Compared to unprocessed foam beads, the detached particles are slightly smaller. Some also show pressure marks caused by the compaction during the RF welding process.

Now there are two potential recycling options for the detached particles. In a second cycle, these particles could be recoated and processed into new geometries by RF welding. In this case, the water in which the coating was dissolved could be used as the basis for a new coating solution. This would ensure the complete recyclability of all materials used in cycles. Thus, compared to the fully welded geometries made out of particle foams, a significantly easier recycling of the used materials is possible. The second recycling option is to use the detached particles as pure post-consumer material for the manufacturing of other products.

## 4. Summary

It could be shown that the generation of back-dried PVA coatings is suitable as a processing aid for RF welding of foam particles with a low dielectric loss factor such as ePP. Compared to the analyzed ePP, the applied coating material shows high permittivity and dielectric loss values for the used frequency of 27.12 MHz. For all investigated coatings, a sufficient heat production compared to uncoated particles was achieved. 

Since the RF-heating process parameters were kept constant for all materials, the differences in the heating rates correspond to the results of the dielectric characterization. Thus, both a higher degree of saponification and the introduction of the plasticizer glycerol lead to higher permittivity and dielectric loss values, which favor faster heating. For the temperatures reached in the heating trials, the bonding of the produced plates is obtained by the fusion of the PVA coatings, which adhere to the surface of the foam beads. Furthermore, the melting behavior of the coating materials seems to influence the number of joints formed between the particles. While a greater number of OH- side groups is reflected in higher melting points, the peak melting point can be reduced by the use of the plasticizer glycerol. Accordingly, glycerol can be used to specifically adjust both the melting behavior and the dielectric properties of the coating material used.

Despite the absence of a welding between the ePP particles, the plates resisted compression for up to 20% of the initial sample height. Independent of the coating composition, the measured stress–strain curves of the selected coatings were comparable. Since the compound is formed exclusively by the water-soluble coating material, the particles can be easily separated in hot water, which allows a complete separation of the used materials by type.

## 5. Outlook

Part of the current and planned research is the variation of the welding temperature inside the foam part as well as the influence of the mold temperature on the surface appearance. Additionally, the presented coating method should be transferred to other foam materials such as ePS, ePET, or ePLA. These materials show comparable low permittivity and dielectric loss factor to ePP and are therefore not processable inside RF welding without processing aids. Further enhancements of the heating behavior might be achievable by a modification of the coating materials with biological materials such as cellulose and starch or with other plasticizers in order to further increase permittivity and the dielectric loss factor. Finally, the influence of recycling cycles on the weldability of the recoated foam beads combined with the resulting mechanical and thermal properties should be investigated. 

In preliminary tests, it has already been observed that by increasing the welding temperature during the RF process, a welded joint between the ePP particles can be achieved. Similar to the production in the steam chest molding process, this leads to a permanent connection between the ePP particles. The effects on the mechanical properties in comparison to the joined geometries presented in this work need to be analyzed. Finally, a comparison has to be made with specimens from the steam chest molding process, which is the standard process for the production of welded particle foam components.

## Figures and Tables

**Table 1:** Composition of aqueous solution and solid samples of the glycerol-modified materials.

PVA Type/Glycerol Content	Aqueous Solution (vol-%)			Back-Dried PVA (vol-%)	
	Water	PVA	Glycerol	PVA	Glycerol
3-85/0	90	10	0	100	0
3-85/5	90	9.5	0.5	95	5
3-85/10	90	9	1	90	10
3-85/15	90	8.5	1.5	85	15
3-85/20	90	8	2	80	20
3-85/25	90	7.5	2.5	75	25

**Table 2:** Main process parameters for injection molding of PVA and ePP.

	PVA/PVA-Glycerol	ePP
Mold temperature (°C)	50	60
Cylinder temperature (°C) inlet → nozzle	220–230–235–240	210–215–220–230
Injection speed (cm^3^/s)	20	20
Holding pressure (bar)	300	200

## Data Availability

The data presented in this study are available on request from the corresponding author.
